# How Early-Life Gut Microbiota Alteration Sets Trajectories for Health and Inflammatory Bowel Disease?

**DOI:** 10.3389/fnut.2021.690073

**Published:** 2021-08-06

**Authors:** Feilong Guo, Demin Cai, Yanwei Li, Haotian Gu, Huan Qu, Qiufang Zong, Wenbin Bao, Aoxue Chen, Hao-Yu Liu

**Affiliations:** ^1^Epigenetics and Epigenome Research Institute, Yangzhou University, Yangzhou, China; ^2^Department of General Surgery, Jinling Hospital, School of Medicine, Nanjing University, Nanjing, China; ^3^Department of Medical Cell Biology, Uppsala University, Uppsala, Sweden; ^4^College of Animal Science and Technology, Yangzhou University, Yangzhou, China; ^5^Department of Psychiatry and Psychotherapy, University Hospital, Ludwig-Maximilians-Universität München, Munich, Germany

**Keywords:** early-life intervention, gut microbiota, probiotics, weaning, inflammatory bowel disease

## Abstract

Inflammatory bowel disease (IBD) is a recurrent chronic inflammatory condition of the intestine without any efficient therapeutic regimens. Gut microbiota, which plays an instrumental role in the development and maturation of the immune system, has been implicated in the pathogenesis of IBD. Emerging evidence has established that early-life events particularly maternal influences and antibiotic treatment are strongly correlated with the health or susceptibility to disease of an individual in later life. Thus, it is proposed that there is a critical period in infancy, during which the environmental exposures bestow a long-term pathophysiological imprint. This notion sheds new light on the development of novel approaches for the treatment, i.e., early interventions, more precisely, the prevention of many uncurable chronic inflammatory diseases like IBD. In this review, we have integrated current evidence to describe the feasibility of the “able-to-be-regulated microbiota,” summarized the underlying mechanisms of the “microbiota-driven immune system education,” explored the optimal intervention time window, and discussed the potential of designing early-probiotic treatment as a new prevention strategy for IBD.

## Introduction

Inflammatory bowel disease (IBD), including ulcerative colitis and Crohn's disease, is a life-long disease once onset. It is characterized by recurrent intestinal inflammation, causes cumulative and progressive damages to the bowel wall, and consequently leads to intestinal dysfunction, obstruction, or perforation that require surgical intervention (1). IBD affects millions of people in North America and Europe, and the incidence is increasing alarmingly with the progression of global urbanization, particularly in the newly industrialized areas, such as Asia (2). Though the last decades have witnessed a great advancement of medicines for IBD, especially the successive launch of different biologics anti-TNF, anti-adhesion agents, and anti-IL-12/23 p40 antibody, etc., a proportion of patients are still not amenable to any medications. Some lose response over time, some have to frequently change drugs due to severe adverse effects, and none of the regimens is available to reverse the intestinal damages (3). Therefore, new therapeutic strategies are in great demand.

Several epidemiological studies have established a positive correlation between the early-life exposures and the future risks of IBD, including the mode of delivery (4), the feeding types (5), the childhood hygiene (6), and the antibiotic use (7). All of which are important environmental factors associated with gut microbiota alterations. In infancy, the gut microbiota is less diverse and resilient and more sensitive to the modulation. The succession of gut microbiota parallels with the development of the gut immune system and directs the health outcome *via* the education of immunity (8). As unstable and sensitive as the infant bacterial community structure is, it also opens up a “window of opportunity” for the associated intervention (8, 9). From this perspective, reducing the environmental incursions or facilitate the gut microbiota equilibrium within an optimal time frame can be a new therapeutic strategy for IBD. In this review, we will first describe the postnatal development of gut microbiota and the intestinal immune system, taking macrophages (MP) as an example. Then, we will summarize current evidence depicting the “window of opportunity” for the gut microbiota modulation, and finally discuss the potential of designing early probiotic treatment as a new prevention strategy for IBD.

## Gut Microbiota of Newborns

The mammalian gastrointestinal (GI) tract accommodates the highest density of the microbial community on earth, more precisely in the distal part where up to 100 trillion microorganisms inhabit. It is far beyond the number of eukaryotic cells of the host that holds itself (10). A healthy adult intestine harbors 100s of bacterial species, including the dominant genera *Bacteroides, Clostridium, Prevotella, Faecalibacterium, Eubacterium, Ruminococcus, Peptococcus, Peptostreptococcusm*, and *Bifidobacterium*, which are affiliated to the four major phyla, i.e., *Bacteroidetes, Firmicutes, Proteobacteria*, and *Actinobacteria* (11, 12). Despite the considerable variation among individuals detected in the microbiome, Arumugam et al. (10) have identified three distinct clusters of gut microbiota driven by discriminative genera such as enterotype1-*Bacteroides*, enterotype2-*Prevotella*, and enterotype3-*Ruminococcus*, which applies to the global microbiome of human beings and is generally stable over time and in geography (13).

Gut microbiota shows an age-dependent succession difference. Unlike the stable enterotypes in adults and the deterioration occurring in old age (13), the gut microbial ecosystem of newborns is characterized by rapid changes in bacterial abundance, diversity, and large interindividual variability of community composition (14). Conventionally, the human GI tract is first colonized by bacteria from the immediate environment. Often, by the maternal vagina and feces-associated microbes including *Lactobacillus* and *Prevotella* in infants of vaginal birth, or by bacteria from maternal skin and the surroundings represented by *Staphylococcus* in babies of cesarean delivery (15). Of note, recent studies have also reported the microorganisms detections in the placenta (16) or amniotic fluid (17). Nevertheless, facultative anaerobes, predominantly *Escherichia coli* and other bacteria that belong to *Gammaproteobacteria* are the first gut colonizers. Subsequently, the oxygen in the intestinal microenvironment is exhausted by these bacteria and becomes anaerobic that favors the growth of strict anaerobes such as *Bifidobacterium, Clostridium*, and *Bacteroide*s, which constitute an early community of low diversity. The aerobic to anaerobic transition seems to occur very rapidly, as the obligate anaerobes can be detected to occupy the intestine at the first week after birth (18). Thereafter, the *Bifidobacterium* genus presents its diversity and prevalence in the gut microbiome of young infants at the age of 7–42 days (19–21). Given that, human milk is enriched with non-digestible human milk oligosaccharides (HMOs) and the infant *Bifidobacteria* are proficient at utilizing HMOs as the sole carbon source, by dedicating a sizable fraction of the genome to encode the proteins associated with the HMOs metabolism (12). Finally, the introduction of solid foods during weaning (at about 6 months old) primes the infant gut microbiota to adapt to an adult diet, which results in a significant increase in the bacterial community diversity and complexity, accompanied by climbing of *Bacteroidetes* and a decline of *Bifidobacterium* (22). However, a recent study suggests that the cessation of breastfeeding, rather than the solid food introduction, determines the maturation of the microbiome into an adult-like phenotype. It is usually around 1 year of age for human beings, thereafter, the gut microbiota becomes relatively resilient and stable at the age of 3 (8, 14). Notably, two longitudinal cohort studies of Spanish and Swedish infants revealed an overall directionality of gut microbiota changes toward their mothers, both compositionally and functionally within the first 12 months (18, 23).

## Intestinal Macrophage and Early Microbial Imprinting

Constantly exposed to the extremely complicated gut microbiota, the intestine also prepares itself with the largest compartment of the immune system (24, 25), which consists of the organized lymphoid tissue [Peyer's patches (PPs), solitary isolated lymphoid tissues (SILTs), and the draining lymph nodes], along with the scattered effector cells distributed in the lamina propria (LP) and the epithelium (26). Interestingly, although partially programmed before birth, the LP and the epithelium are devoid of mononuclear cells at birth (26).

Among the effector immune cells, the intestinal MPs are the most plastic population. Located directly underneath the surface epithelium in the LP, they serve as sentinels of the first line of defense, are responsible for the bacterial antigen presentation, and discriminating the innocuous agents from the pathogenic insults in the gut lumen, and clearing apoptotic and senescent epithelial cells (27). MP are important players in maintaining gut homeostasis and contribute to IBD pathogenesis (28). Discrete populations of MPs are also found in the submucosa and muscularis mucosa throughout the GI tract, together which exhibit a variety of functions to ensure the proper immune reactions (29). Apart from being highly phagocytic and producing TNF-α, IL-1, and IL-6 in response to inflammation, the intestinal MPs also mediate mucosal tolerance by secreting the anti-inflammatory cytokine IL-10, thereby promote the regulatory T-cell population (28, 30), suggesting a case of innate immunity that controls adaptive immunity. The introduction of the “macrophage-waterfall” of Ly6C^hi^ monocytes, which develop progressively into CX_3_CR1^hi^-MHCII^hi^ resident cells based on CD64 expression, makes it possible to more accurately identify the intestinal resident MPs (28). The gut MPs represent a mixed population, constantly replenished by the blood monocytes originated from the bone marrow (29). During the neonatal period, the MPs derived from embryo and yolk sac predominate the cell pool (31), but only the latter is found to be replaced by cells from a series of development including the chemokine receptor CCR2-dependent recruitment of Ly6C^hi^ monocytes, downregulation of Ly6C, and upregulation of F4/80, CD64, and CX_3_CR1 expressions, as well as obtaining the MHCII phenotype. This process-driven largely by the microbiota, occurs during weaning and peaks during the third week of life in mice, whereas germ-free mice are found to have fewer MP in the gut wall, and its turnover can be suppressed by antibiotics (29).

The phenotypic and functional identity of the MPs is imprinted by the local environment. Microbiota as a whole is an important stimulus for monocyte-MP differentiation (32). While the number and the subsets diversity change during intestinal inflammation and infection (27, 29). It is suggested that the gut MPs are susceptible to environmental (re)programming, even with the matured phenotype. There is a causal link between the intestinal MPs and IBD, where the altered monocyte-macrophage differentiation impairs the resolution of intestinal inflammation in patients with IBD, leading to the chronic relapse in individuals with the genetic predisposition (33). On the other hand, we postulate in this study that the targeted MPs polarization of the intestinal microenvironment, by therapeutic strategies like the probiotic usage at an early stage, may induce sustained intestinal immune protection. This is supported by a recent study by Danne et al. (34) that *Helicobacter hepaticus-*polysaccharides induced an anti-inflammatory MP response.

## Critical Time Windows Exist

As discussed above, the microbial colonization of the gut of an infant represents the *de novo* assembly of a bacterial community with functional attributes similar to adults. It usually takes 2–3 years for humans and 4–6 weeks for mice (22). Important changes are witnessed during weaning when the solid food is gradually introduced to a suckling mammal while withdrawing the milk of its mother (9, 35). This leads to a “weaning reaction” of the immune system against the microbiota alterations. A proper “weaning reaction” is associated with changes in the global gene expression in the intestine, such as genes encoding defensins, chemokine receptors, and mucins. Furthermore, commensal microbiota induces gene upregulations of the pro-inflammatory cytokines TNF-α and IFN-γ during weaning at 3 weeks of age in mice (9). An elevation of IL-1β expression was also observed in 21-days old rats at weaning (36). In contrast, the inhibition of “weaning reaction” in mice using antibiotics led to a “pathological imprinting” of the immune system, which cannot be reversed after weaning (9).

It is, therefore, instrumental that the microbiota establishes its mutually beneficial cohabitation with the host in the time window of early life, whereas the perturbations may result in potentially persistent immune abnormalities (8, 37). Several genome-wide association analyses and large-scale cohort studies reveal that exposure to antibiotics in childhood especially during the first year of life is associated with increased susceptibility to IBD and allergy (7, 37, 38). Furthermore, a meta-analysis demonstrates that breastfeeding is associated with lower risks of IBD, with even lower disease incidence in infants under 12 months breastfeeding regimen than 6 months (39). A study employing surrogate markers of childhood hygiene (e.g., fewer siblings and living in urban areas) revealed that individuals raised in a sanitary environment are at a higher risk of IBD later in life (6). In contrast, children raised close to farm animals develop less inflammation and allergies, which supports the “hygiene hypothesis” (40). In germ-free mice, the immunological abnormality can be reversed by introducing the commensal bacteria from a healthy counterpart but only as an early-life intervention (41). Furthermore, Cox et al. (35) found that temporal antibiotics administration causes a transient microbiota perturbation in mice infancy but induces a long-term metabolic phenotype. These results highlight the importance of the early-life microbial perturbations vs. the early-life intervention, with the emphasis on gut microbiota modulation (8, 37). There is a saying that “who started the trouble should end it.” It appears that several aspects of immune development are indeed more permissive to the microbial-mediated changes during early life, and that specific bacterial taxa are crucial in these interactions.

## The Potential of Probiotics For Early Intervention

Because gut microbiota perturbation is one of the major causes of IBD and the modulation is a goal, it is relevant to consider using probiotics as a therapeutic strategy. Indeed, live microorganisms given personally produce health benefits, are defined as “probiotics” and have intrigued humans for centuries (42). In particular, the probiotic effects in IBD treatment have been studied to reverse the dysbiosis-associated inflammation (43). Although having been added widely in snacks, drinks, infant formulas, and consumed as health supplements, the clinical use of probiotics as therapeutics and their effects are still less demonstrated (42). The protective effects in animal models of IBD and *in vitro* studies were robust as seen by several conventional probiotic strains, e.g., *Bifidobacterium infantis, Lactobacillus plantarum*, and *Lactobacillus reuteri*, as well as verified by novel strains *Lactobacillus helveticus* PI5 and *Lactobacillus salivarius* LA307 (44). All of them point to the efficacy and proficiency in strengthening the intestinal epithelial barrier and improving the immune functions and the microbiome (45). However, their clinical effects with patients with IBD were poorly verified. In a meta-analysis study, no effect was observed on probiotic-induced remission or prevention of relapse in Crohn's disease, whereas some beneficial effects were shown in patients with colitis (46).

One enigma is whether or not the probiotic bacteria could colonize the intestine by peroral administration, if so for how long they could stay in place after the administration is removed. This would challenge the actions of probiotics when regarded as contact-dependent (47). As aforementioned, the established microbiota in adulthood remains quite stable albeit the fluctuation under drastic changes (10). A recent study demonstrates that human targeted probiotics exhibited low-level mucosal colonization in SPF mice, due to resistance from the indigenous microbiome (48). Several studies found a decline of probiotic bacteria detection in the fecal microbiota shortly after the administration cessation (49, 50). In human beings, the probiotics differentially affect the “permissive” and “resistant” individuals, which features a personal effect of combined factors including microbiota variation and host gene signatures associated with immune responses and metabolism (48). This could be a possible explanation for the ambiguous effects achieved in clinical trials of IBD-probiotic treatments. In both humans and mice, the probiotics are seen to induce changes in microbiome and host GI transcriptome, despite the limited colonization (48). The mechanisms behind this and the causality of the microbiome in IBD onset warrant further studies. In this regard, approaches combining high-resolution single-cell RNA sequencing/scRNA-seq of inflammatory lesions with the clinical characterization of patients with IBD may provide deeper insights (51). Interestingly, using scRNA-seq, Aschenbrenner et al. (52) revealed that bacteria exposure induces functional IL-10 resistance in monocytes and a hyperinflammation-associated IL-23 production in patients with severe ulcerative colitis.

Surprisingly, several recent studies have described enhanced probiotic colonization within or post-antibiotic treatment, suggesting that probiotics were more likely to act in the “microorganism less abundant” niches (53, 54), as a property that can be found in the gut microbiota during early life. Furthermore, Schultz et al. (55) have shown that administration of *Lactobacillus rhamnosus* GG to pregnant women causes infantile colonization for up to 24 months and increased the bifidobacteria diversity in neonates. During the postnatal period, *L. reuteri* DSM 17938 treatment is shown to promote lactobacilli growth while inhibiting that of *E. coli* in infants with colic (56). This is confirmed to be effective as an infantile colic intervention. The probiotic treatment is also considered well-tolerated and safe in preterm infants, to prevent necrotizing enterocolitis and all-cause mortality (57). However, there are very few longitudinal studies concerning the probiotic intervention in infants and track their IBD susceptibility growing up. It is recently shown that infants born to IBD mothers exhibited altered gut microbiome from the first week after birth to at least 3 months old, causing aberrant adaptive immune responses (58). This again underlines the urgency of early microbiota modulation for IBD prevention.

We suggest that a better understanding of the gut microbiome signature in infant exposure to maternal influences changes of diets and medications using the readily available next-generation sequencing tools could help to develop predictive markers and guide the selection of the probiotic strain fitted for the potential early-life intervention. For instance, giving probiotic *Bifidobacterium* strains to the IBD mother-delivered babies, who displayed a depletion of bifidobacteria in the first week of life (58) to promote their health, or use a combination of probiotic strains to enrich the gut microbiota with low diversity, like in the cesarean section-delivered newborns (15) and formula-fed infants (39). For early antibiotic perturbations associated with the increase of opportunistic pathogens, a probiotic designed to target the corresponding bacterial taxa (e.g., *Gammaproteobacteria*) should be considered. In addition, probiotics can also be tailored for metabolic reprogramming, for instance, to restore a lipid dysregulation caused by dysbiosis during a disrupted “weaning reaction.” For those breast-fed, virginally delivered, full-term healthy babies, who are not exposed to antibiotics but could still suffer from IBD, the probiotic strains should be selected according to the normal gut microbiota succession trend discussed above. For example, with the probiotic strains from *Actinobacteria* given before weaning and those from *Firmicutes* afterward. Meanwhile, the characteristics of the gut microbiome in adult patients with IBD should be used as a reference for early intervention.

## Conclusion

**Figure 1:**
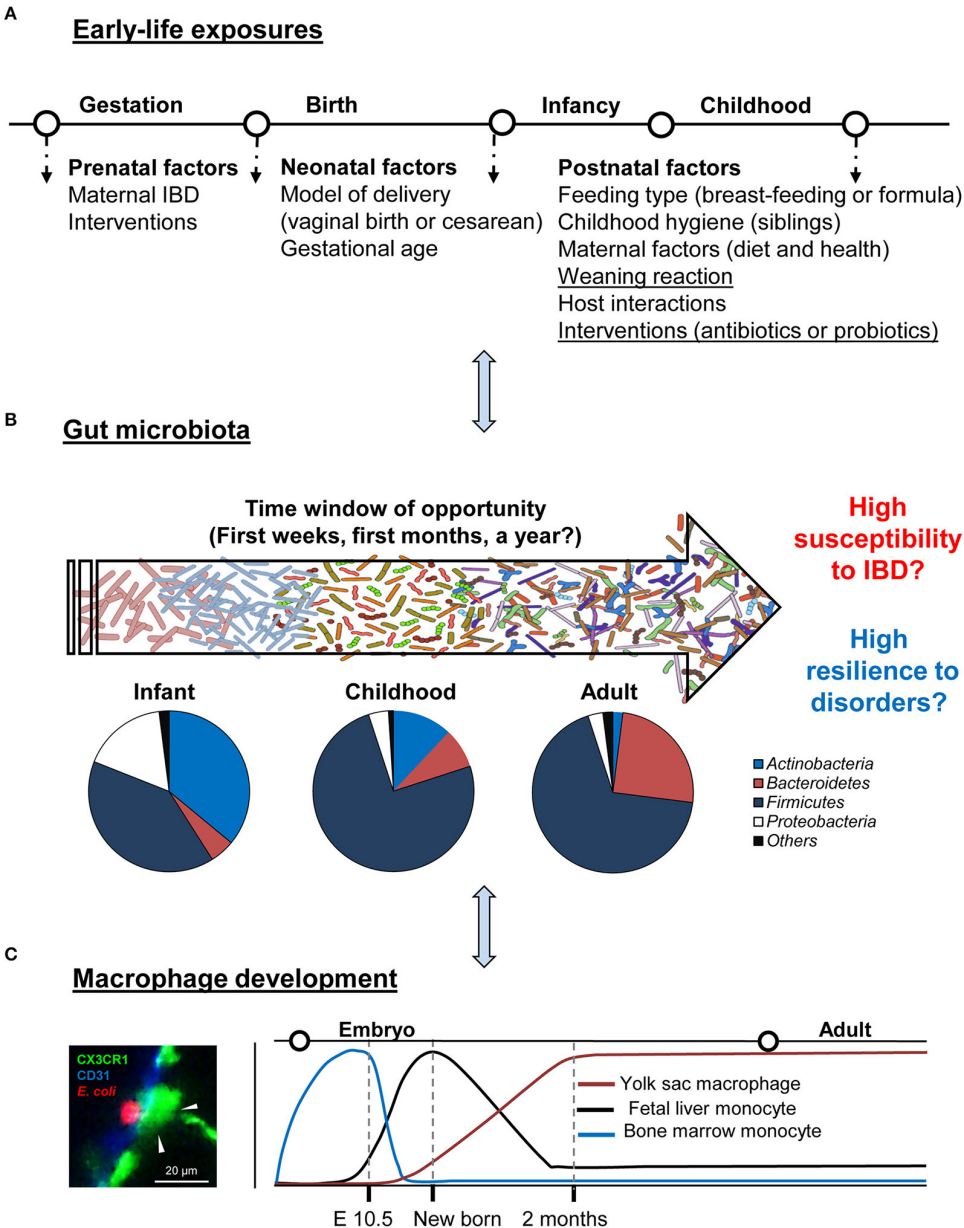
Critical time window: gut development and regulation. **(A,B)** Schematic summary of early-life environmental factors that impact the gut microbiota establishment of an individual and drive its phenotype toward health or IBD. Typical phylum-level of microbiota composition in healthy individuals in different life stages and the window of opportunity for possible modulation. **(C)** Gut resident macrophage ontogeny in the steady-state. A representative image of colon macrophage (green), interacting with *Escherichia coli* (red, left panel).

Because gut microbiota is implicated in human and animal health and disease, an essential goal of this review is to better understand the assembly and community composition of the microbiota with a special emphasis on the early-life period. Unlike the adult microbiota, which is relatively resistant to perturbations and stable over time, the gut microbial ecosystem of the newborn is characterized by rapid changes in bacterial community composition, with lower diversity and lower complexity. Therefore, a convergence in gut microbiota succession, environmental stimuli for the time being, and maturation of the immune system decide the disease susceptibility in later life (Figure 1). Especially for IBD that is life-long and is featured by recurrent chronic intestinal inflammation and dysregulation of the immunity toward commensal bacteria. Since the immune system is more permissive to microbial-mediated changes during the early-life period, specific probiotic bacteria could be the key to the potential modulation. This early-life period (i.e., from born till the microbiota reach an adult phenotype) opens up an exciting “window of opportunity.” However, it should be carefully defined, categorized, and evaluated. Stable and resilient gut microbiota in an adult but with pathological imprinting can be detrimental in many aspects. This may explain why there are “permissive” and “resistant” patients with IBD to medications.

## Author Contributions

H-YL, WB, and FG conceived this review. FG, DC, AC, YL, HG, HQ, and QZ wrote the manuscript. H-YL, WB, and AC supervised and approved the final version. All the authors have read, revised, and approved the final manuscript.

## Conflict of Interest

The authors declare that the research was conducted in the absence of any commercial or financial relationships that could be construed as a potential conflict of interest.

## Publisher's Note

All claims expressed in this article are solely those of the authors and do not necessarily represent those of their affiliated organizations, or those of the publisher, the editors and the reviewers. Any product that may be evaluated in this article, or claim that may be made by its manufacturer, is not guaranteed or endorsed by the publisher.
