# Corrigendum: How Early-Life Gut Microbiota Alteration Sets Trajectories for Health and Inflammatory Bowel Disease?

**DOI:** 10.3389/fnut.2021.760443

**Published:** 2021-09-15

**Authors:** Feilong Guo, Demin Cai, Yanwei Li, Haotian Gu, Huan Qu, Qiufang Zong, Wenbin Bao, Aoxue Chen, Hao-Yu Liu

**Affiliations:** ^1^Epigenetics and Epigenome Research Institute, Yangzhou University, Yangzhou, China; ^2^Department of General Surgery, Jinling Hospital, School of Medicine, Nanjing University, Nanjing, China; ^3^Department of Medical Cell Biology, Uppsala University, Uppsala, Sweden; ^4^College of Animal Science and Technology, Yangzhou University, Yangzhou, China; ^5^Department of Psychiatry and Psychotherapy, University Hospital, Ludwig-Maximilians-Universität München, Munich, Germany

**Keywords:** early-life intervention, gut microbiota, probiotics, weaning, inflammatory bowel disease

In the published article, there was an error in affiliation 1. Instead of “Department of General Surgery, Jinling Hospital, School of Medicine, Nanjing University, Nanjing, China,” it should be “Epigenetics and Epigenome Research Institute, Yangzhou University, Yangzhou, China.” There was an error in affiliation 2. Instead of “Department of Medical Cell Biology, Uppsala University, Uppsala, Sweden,” it should be “Department of General Surgery, Jinling Hospital, School of Medicine, Nanjing University, Nanjing, China.” There was an error in affiliation 3. Instead of “Department of Animal Nutrition, College of Animal Science and Technology, Yangzhou University, Yangzhou, China,” it should be “Department of Medical Cell Biology, Uppsala University, Uppsala, Sweden.” And there was an error in affiliation 4. Instead of “Epigenetics and Epigenome Research Institute, Yangzhou University, Yangzhou, China,” it should be “College of Animal Science and Technology, Yangzhou University, Yangzhou, China.” Accordingly, the placement of 1, 2, 3, and 4 has been updated.

In the published article, there was an error regarding the affiliations for Feilong Guo. As well as having affiliations 2 and 3, he should also have affiliation 1 “Epigenetics and Epigenome Research Institute, Yangzhou University, Yangzhou, China.”

The authors apologize for this error and state that this does not change the scientific conclusions of the article in any way. The original article has been updated.

## Publisher's Note

All claims expressed in this article are solely those of the authors and do not necessarily represent those of their affiliated organizations, or those of the publisher, the editors and the reviewers. Any product that may be evaluated in this article, or claim that may be made by its manufacturer, is not guaranteed or endorsed by the publisher.

